# Case of Poisoning by Lead in Maccouba Snuff

**Published:** 1843-11

**Authors:** 

**Affiliations:** Copenhagen (Zeitschrift fur die Gessammte Medicin)


*Case of Poisoning by Lead in Maccouba Snuff.* By Professor Otto, of Copenhagen (Zeitschrift fur die Gessammte Medicin).—The following cases have excited universal attention at Copenhagen. Dreyer, highly esteemed as a scholar and a botanist, has enjoyed general good health until the autumn of 1841, when he was seized with the common symptoms of dyspepsia, and with costiveness and a muddy complexion. As he led a sedentary life, exercise was advised and bitter and laxative pills were prescribed. After this, he felt a little better, but just before Christmas, was seized with uncommonly severe colicky pains. His pulse was natural, tongue clean, and the abdomen not painful on pressure. The bowels had been open on the previous day. Enemas containing a grain of opium gave relief. Even then, the suspicion of lead being the cause, was excited, and Dr. Ahrensen supposed that the red wine prescribed, might contain it, but this was found not to be the case.

The improvement did not long continue, the bowels remained costive, the colicky pains returned with cardialgia. He had four of these attacks during the winter, which were removed, partly by purgatives and partly by opiates. In one of these only, was there quickness and tenseness of the pulse, with tenderness of the abdomen, and for this he was bled, and calomel was added to the opium. During the last attack, a very large quantity of round hard fæces were discharged, and after this the patient felt very well. In a week however, he was attacked with violent headache and this ended in a comatose state. In spite of all remedies used, he died at the end of two days, and about four months after the first attack of colic.

On dissection, every part was found healthy in the abdomen, except a trifling enlargement of the colon, probably caused by the frequent clysters; the brain too was healthy, except a slight injection of the membranes.

The deceased was much lamented, but his death had been almost forgotten, when his friend Dr. Bramsen read in a journal that red Maccouba snuff was sometimes adulterated with red lead. On this, he imagined that his deceased friend, who took a great deal of this snuff, might have been poisoned by it. He therefore bought some at the shop where the deceased used to buy his and found that it was mixed with a number of large and small grains, which looked like minium, and on chemical examination, he discovered that the snuff contained from sixteen to twenty per cent. of lead.

In the case of a young physician still alive, who is a great snuff-taker and long used the same snuff as the deceased patient, there has been an affection of the abdomen, characterized by colicky pains and obstinate costiveness. This has continued for a year, and he has become very thin. As he has now left off taking the snuff, it is to be hoped that he will be cured.—*Amer. Journ. of the Med. Sci.*, from *Lond. Med. Gaz.*, April, 1843.

